# Conduction System Pacing: Have We Finally Found the Holy Grail of Physiological Pacing?

**DOI:** 10.17925/HI.2023.17.2.3

**Published:** 2023-12-01

**Authors:** Myriam Kaddour, Haran Burri

**Affiliations:** Cardiac Pacing Unit, Cardiology Department, University Hospital of Geneva, Geneva, Switzerland

**Keywords:** Biventricular pacing, cardiac resynchronization therapy, conduction system pacing, His bundle pacing, left bundle branch pacing, physiological pacing, right ventricular pacing

## Abstract

The late fifties are considered a high point in the history of cardiac pacing, since this era is marked by the first pacemaker implantation, which has since evolved into life-saving therapy. Right ventricular apical and biventricular pacing are the classic techniques that are recommended as first-l ine approaches for most indications in current guidelines. However, conduction system pacing has emerged as being able to deliver a more physiological form of pacing and is becoming mainstream practice in a growing number of centres. In this review, we aim to compare traditional pacing methods with conduction system pacing.

For decades, right ventricular pacing (RVP) has been the leading pacing technique and has been proven to be effective in treating patients with symptomatic bradycardia. However, dyssynchrony caused by non-physiological ventricular activation results in pacing-i nduced cardiomyopathy occurs in approximately 15% of patients with >20% ventricular pacing after 5 years.^1^ Pacing of the right ventricular septum has not been shown to be superior to apical pacing.^2^ These findings have led to the quest for new methods to avoid the harmful effects of RVP. Cardiac resynchronization therapy (CRT) with biventricular pacing (BiVP) was introduced to treat heart failure in patients with ventricular dyssynchrony resulting from intra-ventricular conduction disorders and is one of the success stories of ventricular pacing. Limited data show that this form of pacing may also be used to avoid cardiac dysfunction in patients requiring ventricular pacing who have preserved baseline ejection fraction.^3^ However, BiVP has never become first-l ine therapy for all-comers requiring ventricular pacing, as implantation may be complex and the systems come at a supplementary cost. Conduction system pacing (CSP) has more recently emerged as an alternative to RV and BiVP to provide truly physiological pacing in a simple, effective and economical manner.

The first description of His bundle pacing (HBP) dates back to 1967 in canine hearts.^4^ In 2000, Desmukh et al. published the first article on HBP in humans, which laid the foundation for subsequent research in this area.^5^ Fifteen years later, Huang et al.^6^ pioneered left bundle branch area pacing (LBBAP) in a patient with heart failure and complete left bundle branch bloc and showed feasibility and positive outcomes after 1 year of follow-up. Since the last decade, CSP adoption has grown steadily (see *Figure 1*) and is predicted to dominate over conventional pacing in the years to come according to a recent European Heart Rhythm Association (EHRA) survey.^7^

## Implantation technique and pacing parameters

The recommended implantation technique for RVP has been outlined in an EHRA consensus document and will not be elaborated here.^8^ BiVP has been considerably simplified by the advent of guiding catheters which have facilitated canulation of the coronary sinus and by quadripolar leads,^9^ as well as with active fixation, which have reduced dislodgment rates and requirement for re-i ntervention.^10^ Successful lead implantation is approximately 98% with current tools, and failures mainly being attributed to lack of suitable coronary sinus tributaries.^11^ Nevertheless, approximately 80% of patients have the coronary sinus lead placed in a lateral or postero-l ateral position, which are the typically targeted tributaries.^11^ Furthermore, delivery of CRT may be hampered by phrenic nerve capture and high capture thresholds.

CSP implantation has been standardized in a recent EHRA consensus document which provides a framework for the procedure.^12^ CSP implantation requires recording of a 12-l ead electrocardiogram (ECG) to recognize conduction system capture using specific criteria,^12,13^ ideally with an electrophysiology recording system. Dedicated 3D-shaped delivery catheters facilitate lead placement for HBP and for LBBAP. However, current pacing leads are not specifically designed for CSP implantation. Technical difficulties remain, such as penetration of the central fibrous body for HBP, or penetration of fibrotic interventricular septa, and prevention of micro/macro lead dislodgement within the tunnel drilled by the LBBAP lead. Implantation success rate for HBP has been reported to be 93% for patients with nodal atrioventricular block and 76% for those with infra-nodal block.^14^ In the randomized His-Alternative study, which included patients with heart failure with left bundle branch block, implantation success rate was higher for BiVP than for HBP (96% versus 72%, respectively), mainly due to the inability to correct the intraventricular conduction disorder.^15^ Success rate for LBBAP implantation has been reported to be 92% for bradycardia indications and 82% for heart failure indications in the multicentre European MELOS registry,^16^ which included 2,533 patients (the largest LBBAP series reported to date). These figures include the learning curve, which is approxiamately 50 patients in operators with previous experience with HBP.^17,18^

**Figure 1:**
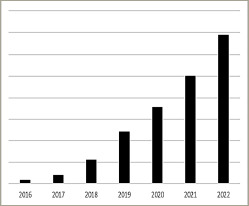
Sales of the Medtronic 3830 lead in Western Europe*

*Evolution of sales of the Medtronic 3830 lead in Western Europe (actual numbers not shown), reflecting adoption of conduction system pacing as this is the lead that is principally used for these procedures.

*The growth between 2016 and 2022 is approximately 35-fold. Data courtesy of Medtronic.*

Compared with RVP, implantation duration of HBP is longer (by approxiamately 15 minutes on average), with a higher rate of lead revisions^19^; LBBAP is also longer (by approxiamately 26 minutes on average) but with comparable electrical parameters and rate of lead revision.^20^ Compared with BiVP, implantation duration is on average about 10 minutes longer with CSP with lower capture thresholds.^21^ QRS duration is shorter with CSP compared with RVP and BiVP.^19–21^

In a recent European survey,^22^ CSP implanters favour LBBAP over HBP for most indications, mainly due to superior electrical parameters and perceived ease of implantation.

## Complications

Some complications, such as pneumothorax, pocket haematoma, device infection, cardiac arrhythmias and lead dislodgment, are common to all forms of pacing. Lead-related tricuspid regurgitation is another complication that is increasingly recognized but reported with variable incidence due to the retrospective nature of most studies and non-systematic evaluation before and after implantation. In a prospective study randomizing RV apical, RV septal and coronary sinus pacing, new moderate or severe tricuspid regurgitation was observed in 6% of patients after 1 year of follow-up.^23^ This was due to impingement of the septal leaflet or interference with leaflet coaptation (including prolapse of a coronary sinus lead). Other described mechanisms for this complication are impairment of the valve closure due to scar, thrombosis or valve perforation.^24^ LBBAP implantation has also been associated with tricuspid regurgitation, especially if the lead is implanted in a basal position.^25^ Conversely, HBP is associated with an improvement in tricuspid regurgitation.^26^ This may be due to improved synchrony of cardiac function. HBP has also been associated with an improvement of mitral regurgitation due to reduction of left ventricular volumes and increased contractility.^27^ An additional consideration is absence of interference with valve function with HBP leads placed on the atrial aspect of the tricuspid valve or in the commissure between the septal and anterior leaflets. Regarding LBBAP, in patients with non-i schemic cardiomyopathy and left bundle branch block, a significant improvement of functional moderate to severe mitral regurgitation was observed.^28^

Cardiac tamponade may occur with RVP (by perforation of the RV free wall) and during coronary sinus lead implantation (due to dissection of the coronary sinus at cannulation, balloon venography or by perforation of the coronary sinus tributaries during lead placement). Phrenic nerve stimulation is an issue with coronary sinus leads, which may compromise delivery of therapy. Direct capture of the diaphragm may complicate apical RVP. There also are complications that are specific to LBBAP, such as lesions of septal coronary vessels with acute coronary syndrome, formation of fistula or septal haematoma.^12^ Acute perforation of the interventricular septum is one of the most frequent complications of LBBAP, occurring in up to 14% of patients.^29^ However, if recognized and corrected at implantation it does not have any consequences. Delayed perforation of the septum occurs in <1% of patients.^16^

A major issue with HBP is poor electrical parameters with high capture thresholds, oversensing of atrial/His potentials (which may lead to inhibition of pacing with asystole) or ventricular undersensing. Rates of lead revision are high, up to 13%.^30,31^ Implantation of backup leads in selected patients has been advocated in pacing guidelines^32^ to mitigate the consequences of these electrical issues. However, device programming can be complex in these situations.^33–35^

There are no data regarding long-term extractability of LBBAP leads, and it is likely that specialized tools will have to be developed to achieve this.

## Clinical outcome

As previously mentioned, a major issue with RVP is pacing-i nduced cardiomyopathy, occuring in approximately one fifth of patients with >20% ventricular pacing after 5 years.^1^

RVP is also associated with an increased risk of atrial fibrillation,^36^ which is lower with CSP.^19^

Currently, there are limited randomized data comparing RVP with CSP. A small randomized cross-over study that included 38 patients with atrioventricular block who received both RVP and HBP (most of whom had para-Hissian pacing), found a significantly greater left ventricular ejection fraction (LVEF) after 12 months of HBP.^37^ Observational data indicate superior outcome in terms of death, heart failure hospitalization or upgrade to BiVP in patients with HBP^38^ or LBBAP^20^ in patients who are paced >20% of the time.

There are more data comparing clinical outcome of BiVP with CSP, with currently four randomized trials evaluating HBP^15,39–41^ and two trials evaluating LBBP.^42,43^ All these trials have a relatively limited population size (30–70 patients) but show that CSP results in a narrower QRS with similar or superior improvement in LVEF. In a meta-analysis of 21 studies, CSP was associated with significantly reduced mortality as well as heart failure hospitalization compared with BiVP.^44^

## Indications and current guidelines

**Figure 2:**
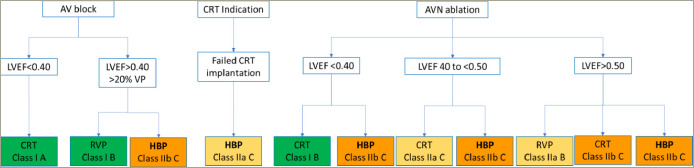
Indications for right ventricular pacing, His bundle pacing and cardiac resynchronization therapy according to the 2021 European guidelines for cardiac pacing.^32^

Traditional RV and BiVP are first-l ine pacing modalities according to the 2021 European Society of Cardiology (ESC) pacing guidelines^32^ mainly because of the lack of randomized trials in the field of CSP as well as limited data on longterm safety. These guidelines only give recommendations for HBP and did not include LBBAP due to limited data at the time of their writing (a summary of the recommendations is shown in *Figure 2*). The American Heart Rhythm Society (HRS) guidelines on physiological pacing have recently been published and include LBBAP at the same level as HBP.^45^ RVP is first-l ine therapy for patients with infrequent pacing.^32,45^ Nevertheless, it could be argued that patients who have a pacing indication for sinus dysfunction may develop atrial fibrillation and may require rate control or that atrioventricular block may worsen over time, which may result in more frequent ventricular pacing. In patients who require frequent ventricular pacing, RVP remains the first-l ine therapy (class 1) in case of LVEF >40% with HBP as a class 2b alternative according to the ESC guidelines.^32^ The HRS guidelines are more detailed in these patients and distinguish LVEF 36–50% and >50%, giving a class 2a indication for BiVP, HBP or LBBP in the former and a class 2b indication for these therapies in the latter categories.^45^ In patients with a “classic” indication for cardiac resynchronization therapy who have left bundle branch block, LVEF ≤35% and New York Heart Association II–IV heart failure, BiVP remains the first-l ine therapy for class 1 indications in the ESC and HRS guidelines. These indications are likely to evolve with more data from randomized trials in the future.

*AVB = atrioventricular block; CRT = cardiac resynchronization therapy; HBP = His bundle pacing; LVEF = left ventricular ejection fraction; RVP = right ventricular pacing;VP = ventricular pacing.*

Recently, His-optimized and LBBAP-optimized cardiac resynchronization therapy (HOT-CRT and LOT-CRT, respectively) have been introduced to fuse CSP with ventricular pacing, which work in a synergistic and complementary manner.^46^ Using ECG imaging, HOT-CRT in patients with incomplete correction of bundle branch block has been shown to provide significantly reduced left ventricular activation times compared with BiVP, without compromising right ventricular activation (and even improving right ventricular activation time in patients with right bundle branch block).^47^ Clinical follow-up has been shown to be improved with HOT-CRT^48^ and LOT-CRT^49^ but no comparison with BiVP or CSP alone have been published to date.

**Table 1:** Advantages and limitations of right/biventricular pacing and conduction system pacing

RVP/BiVP	CSP
*Advantages*	*Advantages*
Widely used due to long experience Shorter procedural duration Simple operating room setup High success rate Long-term evidence for safety and efficacy Improvement of mitral regurgitation (BiVP) Hard evidence from RCTs	Preserves electrical and mechanical synchrony and ventricular function Mid-term evidence for safety and efficacy Avoidance of tricuspid regurgitation (HBP) Improvement of mitral regurgitation Excellent electrical parameters (LBBAP)
*Limitations*	*Limitations*
Pacing-i nduced cardiomyopathy (RVP) High capture thresholds and phrenic nerve capture (BiVP) Increased incidence of atrial fibrillation (RVP) Risk of tamponade (RVP and BiVP) Increased cost (BiVP) High incidence of non-response to therapy in some patient populations (BiVP)	Requirement for 12-l ead ECG (and ideally a electrophysiological recording system) for implantation High incidence of sub-optimal electrical parameters and requirement for lead revision (HBP) Backup ventricular pacing recommended in selected patients (HBP) Complex programming (HBP with backup lead) Complexity to confirm conduction system capture Complications specific to transseptal route (LBBAP) No data on long-term lead extractability (LBBAP) Lower implantation success rate in patients with infra-nodal block (HBP) or heart failure (LBBAP) Correction of bundle branch block in ~60% of patients (HBP) Limited evidence from randomized trials

*BiVP = biventricular pacing; CSP = conduction system pacing; ECG = electrocardiogram; HBP = His bundle pacing; LBBAP = left bundle branch area pacing; RCTs = randomized controlled trials; RVP = right ventricular pacing.*

## Conclusions

CSP is fast evolving towards mainstream practice in centres worldwide. Implantation technique has recently been standardized,^12^ which along with educational and training programmes as well as evolution in the implantation tools will serve to increase uptake of this pacing modality in the future. The advantages and limitations of CSP compared to “traditional” pacing modalities are shown in *Table 1*. Large randomized controlled trials are currently underway, which should hopefully consolidate indications for this therapy in future guidelines, for the benefit of our patients.
